# Electrical Pulse Stimulation of Cultured Human Skeletal Muscle Cells as an *In Vitro* Model of Exercise

**DOI:** 10.1371/journal.pone.0033203

**Published:** 2012-03-22

**Authors:** Nataša Nikolić, Siril Skaret Bakke, Eili Tranheim Kase, Ida Rudberg, Ingeborg Flo Halle, Arild C. Rustan, G. Hege Thoresen, Vigdis Aas

**Affiliations:** 1 Department of Pharmaceutical Biosciences, School of Pharmacy, University of Oslo, Oslo, Norway; 2 Faculty of Health Sciences, Oslo and Akershus University College of Applied Sciences, Oslo, Norway; Instituto de Investigación Hospital 12 de Octubre, Spain

## Abstract

**Background and Aims:**

Physical exercise leads to substantial adaptive responses in skeletal muscles and plays a central role in a healthy life style. Since exercise induces major systemic responses, underlying cellular mechanisms are difficult to study in vivo. It was therefore desirable to develop an *in vitro* model that would resemble training in cultured human myotubes.

**Methods:**

Electrical pulse stimulation (EPS) was applied to adherent human myotubes. Cellular contents of ATP, phosphocreatine (PCr) and lactate were determined. Glucose and oleic acid metabolism were studied using radio-labeled substrates, and gene expression was analyzed using real-time RT-PCR. Mitochondrial content and function were measured by live imaging and determination of citrate synthase activity, respectively. Protein expression was assessed by electrophoresis and immunoblotting.

**Results:**

High-frequency, acute EPS increased deoxyglucose uptake and lactate production, while cell contents of both ATP and PCr decreased. Chronic, low-frequency EPS increased oxidative capacity of cultured myotubes by increasing glucose metabolism (uptake and oxidation) and complete fatty acid oxidation. mRNA expression level of pyruvate dehydrogenase complex 4 (PDK4) was significantly increased in EPS-treated cells, while mRNA expressions of interleukin 6 (IL-6), cytochrome C and carnitin palmitoyl transferase b (CPT1b) also tended to increase. Intensity of MitoTracker®Red FM was doubled after 48 h of chronic, low-frequency EPS. Protein expression of a slow fiber type marker (MHCI) was increased in EPS-treated cells.

**Conclusions:**

Our results imply that *in vitro* EPS (acute, high-frequent as well as chronic, low-frequent) of human myotubes may be used to study effects of exercise.

## Introduction

Physical training leads to extensive adaptations in skeletal muscles [1]-[4]. Regular physical activity plays central role in both prevention and improvement of many chronic diseases, improvement of life-style and increased life expectancy [2]. However, molecular mechanisms underlying these adaptations are still poorly understood, emphasizing the requirement for a cell culture model resembling training *ex vivo*.

Motor neuron activation of muscle fibres can be replaced by electrical pulse stimulation (EPS) of differentiated skeletal muscle cells (myotubes) in culture [5], [6]. Metabolic and genetic adaptations caused by EPS *in vitro* have previously been described in murine C2C12 cells [7]-[9], in L6 cells [10] and in primary rat skeletal muscle cells [11]. *In vivo,* immediate effects of EPS, such as activation of glucose uptake and glycogenolysis [12] can be clearly distinguished from more profound changes in the metabolic and transcriptional phenotypes of muscles occurring as a result of chronically increased contractile activity evoked by chronic, low-frequency EPS [12]. Likewise, acute changes in skeletal muscle after a single bout of exercise *in vivo* differ considerably from those observed after regular training, which, obviously, confers for the most of the beneficial health effects of exercise *in vivo* [2], [13].

Adaptations of metabolic properties in skeletal muscles after exercise are reflected by both increased mitochondrial content [14], [15] and improved oxidative capacity [4], [16], [17]. Several signalling pathways have been proposed to be involved in the stimulation of mitochondrial biogenesis in a contracting muscle [18]-[20]. Exercise has also been shown to enhance both lipid synthesis and lipid oxidation [20]-[23]. As a consequence of these metabolic adaptations, trained muscle takes more of its required energy from lipids and less from carbohydrate compared to untrained muscle during submaximal work (i.e. work performed below the maximal oxygen utilization capacity) [24]. In addition to fatty acids, trained fibers import and use more glucose than untrained muscle fibers [25], [26]. Glucose transporters 1 and 4 (GLUT1 and GLUT4) are the major glucose transporters in the cell membrane of skeletal muscle cells [27], and regular physical activity improves the ability of insulin to stimulate the uptake of glucose at rest [28]-[30].

Plasticity of skeletal muscles in response to endurance exercise extends beyond the metabolic changes. In human skeletal muscles, three main muscle fiber types, type I (oxidative, slow twitch), IIa (intermediate) and IIx (glycolytic, fast twitch), can be delineated based on histochemical, functional and biochemical properties [31]. Type I fibers are characterized by higher mitochondrial content and increased glucose transport compared to type II fibers, and whole-body insulin-sensitivity has been positively correlated with the proportion of slow-twitch oxidative fibers in humans [32]. Conversion from type II to type I muscle phenotype has been shown in animals [33], but there is little evidence that this type of transition actually happens in adult humans [15], [24]. Chronic, low-frequency EPS *in vivo* has been shown to lead to the transformation of fast-twitch glycolytic muscle fibres into slow-type oxidative fibres [12]. Moreover, skeletal muscle has recently been identified as an organ that produces and releases several cytokines, which are termed “myokines”, among these are interleukins 6, 8 and 5 (IL-6, IL-8 and IL-5) [34]. It has been demonstrated that plasma concentration of IL-6 increases during muscular exercise [35], [36], and IL-6 appears to have positive effects on skeletal muscle glucose metabolism [37], [38]. These findings suggest that also the immune system is affected by physical exercise; however, the implications this might have on the metabolic responses are not yet understood.

We have previously reported effects of acute electrical stimulation on glucose metabolism in cultured human skeletal muscle cells, both at high and low glucose concentrations [39]. In the past years, reports of several EPS models applied to cultured skeletal muscle cells have increased in number, suggesting that there is growing interest in establishing a method that would allow to study cellular mechanisms of exercise under controlled conditions *in vitro* [6]-[9], [11], [40]. However, to our knowledge, no model of EPS has been applied to primary human skeletal muscle cells. Cell culture systems derived from muscle biopsies have been used to study glucose and lipid metabolism over the past 30 years. Differentiated primary human myotubes represent the best available alternative system to intact human skeletal muscle. They have the most relevant genetic background to study human disease as opposed to rodent culture systems, and they also display the morphological, metabolic and biochemical properties of adult skeletal muscles [41], [42].

In the present study, we aimed to develop an *in vitro* model of exercise in cultured human skeletal muscle cells, with main focus on metabolic effects of chronic, low-frequency EPS. This model could be used to study adaptive responses of skeletal muscle cells to different types of contractile activity applied by electrical pulse stimulation (EPS).

## Materials and Methods

### Materials

Dulbeccòs modified Eaglès medium (DMEM-Glutamax™), heat-inactivated fetal calf serum (FCS), penicillin/streptomycin (P/S) and amphotericin B were purchased from Gibco (Gibco, Life Technologies Paisley, UK). BSA (Bovine Serum Albumin) (essentially fatty acid-free), L-carnitine, and Dulbecco’s phosphate-buffered saline (DPBS; with Mg^2+^ and Ca^2+^), oleic acid, extracellular matrix (ECM) gel and HEPES were obtained from Sigma (St Louis, MO). Ultroser G was purchased from Ciphergen (Cergy-Saint-Christophe, France), and insulin (Actrapid®) was from NovoNordisk (Bagsvaerd, Denmark). [1-^14^C]oleic acid (55 mCi/mmol) and D-[^14^C(U)]glucose (5 mCi/mmol) were from NEN Radiochemicals, PerkinElmer (Boston, MA). [^3^H]deoxyglucose (10 Ci/mmol) was from American Radiolabeled Chemicals Inc. (St. Louis, MO). Ecoscint A scintillation solution was from National diagnostics (Hessle, England, UK). Glass bottom plates were from MatTek (Ashland, MA). Protein assay reagens was purchased from BioRad (Copenhagen, Denmark). Phospho-Akt (Ser473) and Akt antibodies were from Cell Signaling Technology (Beverly, MA), OXPHOS human cocktail antibodies were from MitoSciences (Eugene, OR) and Anti-Myosin, slow muscle (MAB1628) was from Millipore (Billerica, MA). MitoTracker®Red FM and Hoechst 33258 were obtained from Molecular Probes, Invitrogen (Carlsbad, CA). NuPAGE® 4–12% (w/v) Bis-Tris Gel, 1 mm×12 well was from Invitrogen (Carlsbad, CA). Citrate Synthase Activity Assay Kit was from Sigma-Aldrich® (St. Louis, MO). Cytotoxicity Detection Kit Plus (LDH) was from Roche Applied Science, Mannheim, Germany. The primers for TaqMan Real Time PCR were provided by Invitrogen (Carlsbad, CA). SYBR green and TaqMan reverse transcription kit reagents were obtained from Applied Biosystems (Warrington, UK). Agilent Total RNA isolation Kit was purchased from Agilent Technologies (Santa Clara, CA). All chemicals used were of standard commercial high-purity quality.

### Ethics statement

The biopsies were obtained with informed written consent and approval by the National Committee for Research Ethics (Oslo, Norway). The research performed in this study was approved, as a part of a larger project, by the National Committee for Research Ethics (Oslo, Norway).

### Human skeletal muscle cell cultures

A cell bank of satellite cells was established from muscle biopsy samples of the *Musculus obliquus internus abdominis* of twelve healthy volunteers (10 females, 2 males), age range 34–70 years (50.9±9 years), body mass index (BMI) range 19.6–29.7 kg/m^2^ (23.9±0.9 kg/m^2^), fasting glucose range range 4.9–6.9 mM (5.2±0.2 mM), and plasma lipids and blood pressure within normal range. Not all donors were used in each experiment. Muscle cell cultures free of fibroblasts were established by the method of Henry et al [42]. Briefly, muscle tissue was dissected in Ham’s F-10 medium at 4°C and dissociated by three successive treatments with 0.05% trypsin/EDTA, and satellite cells were resuspended in skeletal muscle DMEM-Glutamax^TM^ with 2% FCS, 2% Ultroser G, 50 U/mL penicillin, 50 µg/mL streptomycin, 1.25 µg/mL amphotericin B, and no added insulin. The cells were grown on culture wells or flasks coated with extracellular matrix gel [43]. After 1–2 weeks, at ∼80% confluence, growth medium was replaced by DMEM-Glutamax^TM^ with 2% FCS, 50 U/mL penicillin, 50 µg/mL streptomycin, 1.25 µg/mL amphotericin B and 25 pM insulin to induce the differentiation of myoblasts into multinucleated myotubes. The cells were cultured in a humidified 5% CO_2_ atmosphere at 37°C, and medium was changed every 2–3 days. All myotube cultures were used for analysis on day 8 or 9 after the onset of differentiation.

### Electrical pulse stimulation of muscle cells

Multinucleated myotubes grown in ECM-coated 6-well plates were stimulated via carbon electrodes either by applying acute, high-frequency EPS (pulse trains of bipolar pulses 100 Hz for 200 ms given every 5^th^ second, 30 V, for 5–60 min), or by applying chronic, low-frequency EPS (single, bipolar pulses of 2 ms, with 30 V and 1 Hz continuously for the last 24 or 48 h of differentiation period). Culturing medium was changed every 12^th^ h during chronic, low-frequency EPS. Electrical pulses were generated by a muscle stimulator built at the Electronics Lab, Institute of Chemistry, University of Oslo.

### Contents of ATP, PCr and lactate

The myotubes were preincubated for 1 h (37°C, 5% CO_2_) with DMEM-Glutamax^TM^. Medium was then changed to fresh DMEM-Glutamax^TM^ and acute, high-frequency EPS was applied for 5–60 min. After stimulation, the cells were immediately placed on ice, the media were removed, and the cells were washed three times with ice-cold phosphate-buffered saline (PBS) before being harvested in 200 µL of ice-cold perchloric acid (3 M). Analyses of ATP, PCr and lactate contents were performed at the Institute for Experimental Medical Research, Ullevål University Hospital, Oslo. The cells were analyzed for ATP and PCr levels with luminescence spectrometry, and lactate content in cells was analyzed with fluorescence spectrometry as described by Lowry et al. [44].

### Measurement of lactate dehydrogenase **(**LDH**)** in culture media from myotubes treated with chronic, low-frequency EPS

Cytotoxic effect of EPS was determined in a colorimetric assay based on the measurement of lactate dehydrogenase (LDH) activity in the supernatant with the use of Cytotoxicity Detection Kit^PLUS^ (LDH) (Roche Applied Science, Mannheim, Germany). Multinucleated myotubes grown in ECM-coated 6-well plates were stimulated via carbon electrodes by applying chronic, low-frequency EPS continuously for the last 24 h or 48 h of the differentiation, and LDH activity in the supernatant was determined according to the supplier’s protocol. Triton-X-100- lysed cells were used for determination of maximum values.

### Glucose metabolism

**Initial experiments with frequency-dependent deoxyglucose uptake:** The myotubes were starved for 60 min in serum-free DMEM-Glutamax^TM^ (5.5 mM glucose) in a 5% CO_2_ incubator at 37°C. Then, the medium was changed to serum-free DMEM-Glutamax^TM^ with [^3^H]deoxyglucose (1 µCi/mL) +/− cytochalasin B (20 µM), and acute EPS was applied for the first 15 min of the 60 min deoxyglucose uptake period, using frequencies of 2 to 10 Hz. **Deoxyglucose uptake at acute, high-frequency EPS:** The myotubes were starved for 60 min in serum-free DMEM-Glutamax^TM^ (5.5 mM glucose) in a 5% CO_2_ incubator at 37°C. Then, the medium was changed to serum-free DMEM-Glutamax^TM^ with [^3^H]deoxyglucose (1 µCi/mL) +/− insulin (100 nM) and +/− cytochalasin B (20 µM), and acute, high-frequency EPS was applied for 5–60 min. **Deoxyglucose uptake after chronic, low-frequency EPS:** Chronic, low-frequency EPS was applied to myotubes continuously for the last 24–48 h of the differentiation period. After ended EPS, the myotubes were starved for 60 min in serum-free DMEM-Glutamax^TM^ (5.5 mM glucose) in a 5% CO_2_ incubator at 37°C, and then exposed to [^3^H]deoxyglucose (1 µCi/mL) +/− insulin (100 nM) and +/− cytochalasin B (20 µM). Deoxyglucose uptake was measured for 60 min for both acute, high-frequency EPS and chronic, low-frequency EPS. After ended uptake, the cells were immediately placed on ice and washed three times with ice-cold PBS, lysed with 0.05 M NaOH, and radioactivity was counted by liquid scintillation. The protein content of each sample was measured according to Bradford [45]. Non-carrier-mediated uptake was determined in the presence of cytochalasin B and subtracted from all presented values. **Glucose oxidation after chronic, low-frequency EPS:** Chronic, low-frequency EPS was applied to myotubes for 24 or 48 h. Then, the myotubes were incubated with glucose-free DMEM and D-[^14^C(U)]glucose (2 µCi/mL) (1 mL/well) in a 5% CO_2_ incubator at 37°C. After 2 h, 500 µL cell medium was transferred to airtight flasks, and 300 µL of phenyl ethylamine-methanol (1∶1, v/v) was added with a syringe to a center well containing a folded filter paper. Subsequently, 100 µL of 1 M perchloric acid was added to the media through the stopper tops using a syringe. The flasks were placed for a minimum of 2 h at room temperature to trap labeled CO_2_, and radioactivity was counted by liquid scintillation The protein content of each sample was measured according to Bradford [45].

### Fatty acid metabolism after chronic, low-frequency EPS

After 24–48 h of chronic, low-frequency EPS, the myotubes were exposed to 1 mL/well of DPBS supplemented with HEPES (10 mM), NaHCO_3_ (44 µM), [1-^14^C]oleic acid (1 µCi/mL, 0.1 mM), 0.24 mM BSA and 1 mM L-carnitine in a 5% CO_2_ incubator at 37°C. After 2 h, 500 µL of cell medium was transferred to airtight flasks, and 300 µL of phenyl ethylamine-methanol (1∶1, v/v) was added with a syringe to a center well containing a folded filter paper. Subsequently, 100 µL of 1 M perchloric acid was added to the media through the stopper tops using a syringe. The flasks were placed for a minimum of 2 h at room temperature to trap labeled CO_2_. To measure ß-oxidation products (acid-soluble metabolites (ASMs)), aliquots of 250 µL of the cell media were precipitated with 100 µL of 6% BSA and 1 mL of 1 M perchloric acid. After centrifugation (20000 *g*, 10 min, 4°C, Heraues Fresco21 Centrifuge, Thermo Scientific), 250 µL of the supernatant was counted by liquid scintillation. No-cell controls were included and subtracted from all presented values. The cells were placed on ice and washed three times with ice-cold PBS, lysed with 0.05 M NaOH, and cell-associated (CA) radioactivity was counted by liquid scintillation to determine the uptake of oleic acid. The protein content of each sample was measured according to Bradford [45].

### Immunoblotting after chronic, low-frequency EPS

Aliquots of 40 µg cell protein from total cell lysates prepared in Laemmli buffer were electrophoretically separated on NuPAGE® 4–12%(w/v) Bis-Tris Gel (Invitrogen) followed by immunoblotting with antibodies recognizing total Akt kinase [protein kinase B (PKB)], Akt phosphorylated at Ser478, and protein complexes of the electron transport chain (OXPHOS human cocktail containing antibodies against Complex I subunit NDUFB8, Complex II subunit, Complex III subunit core 2, Complex IV subunit II and ATP synthase subunit alpha) and Myosin, slow muscle (MHCI). Immunoreactive bands were visualized with enhanced chemiluminescence and quantified with Gel-Pro Analyzer (version 2.0) software.

### Staining and live imaging of mitochondria and enzyme activity assay after chronic, low-frequency EPS

Myotubes were cultured in ECM-coated 6-well glass bottom plates. Chronic, low-frequency EPS was applied to cultured myotubes for the last 48 h of the differentiation period, and in addition, on day 8 of the differentiation, myotubes were incubated at 37°C and 5% CO_2_ with MitoTracker®Red FM (100 nM) for 15 min to stain mitochondria and Hoechst 33258 (2.5 µg/mL) for 15 min to stain nuclei and washed with PBS in between. Automated image acquisition was performed in culture medium without phenol red with an Olympus ScaňR platform (Olympus IX81 inverted fluorescence microscope) equipped with a temperature and CO_2_-enrichment incubator for long-term live imaging, as described in Hessvik et al [46]. We used a 20X objective and live images were aquired in 25 positions per well and 3 wells per treatment per donor were examined. The background-subtracted maximal intensity projection from 7 images taken in z-direction (1 µm apart) was used for both color channels at each position. Olympus ScaňR software was used for automated image analysis, using edge detection algorithm for object segmentation to quantify the number of nuclei and mitochondrial content(total intensity of MitoTracker®Red) per image. After gating out aggregates and dead cells the results were determined from about 386 images per treatment (average of 39±4 nuclei per image). After 48 h of chronic, low-frequency EPS, citrate synthase (CS) activity was determined spectrophotometrically from cell homogenates prepared from the myotubes according to the supplier’s protocol (Citrate Synthase Activity Assay Kit, Sigma-Aldrich®, St. Louis, MO). Citrate synthase activity in cell homogenates from myotubes stimulated for 48 h was compared to activity in homogenates from unstimulated control myotubes.

### RNA isolation and analysis of gene expression by TaqMan® Real-Time RT-PCR

Cells were harvested and total RNA was isolated by Agilent Total RNA isolation kit according to the supplier’s total RNA isolation protocol. Total RNA was reverse-transcribed with oligo primers using a Perkin-Elmer Thermal Cycler 9600 (25°C for 10 min, 37°C for 1 h 20 min, and 85°C for 5 min) and a TaqMan reverse transcription reagents kit. Two micrograms of total RNA were added per 20 µL of total TaqMan reaction solution. Real-time PCR was performed using an ABI PRISMT 7000 Detection System (Applied Biosystems, Warrington, UK). RNA expression was determined by SYBRT Green, and primers were designed using Primer ExpressT (Applied Biosystems, Warrington, UK). Each target gene was quantified in triplicate and carried out in a 25 µL reaction volume according to the supplier’s protocol. All assays were run for 40 cycles (95°C for 12 s followed by 60°C for 60 s. The transcription levels were normalized to the housekeeping control gene 36B4. Another housekeeping control gene tested, GAPDH, gave similar results as 36B4. Following forward and reverse primers were used at concentration of 30 µM: **36B4** (**acc_no M17885**): F:CCATTCTATCATCAACGGGTACAA, R: AGCAAGTGGGAAGGTGTAATCC; **GAPDH** (**acc_no NM002046**): F: TGCACCACCAACTGCTTAGC, R: GGCATGGACTGTGGTCATGAG; **CPT1b** (**acc_no** : **L39211**): F:GAGGCCTCAATGACCAGAATGT, R: GTGGACTCGCTGGTACAGGAA; **cytochrome C** (**acc_no NM001916**): F: CTGCCAACAACGGAGCATT, R: CGTGAGCAGGGAGAAGACGTA; **PGC-1α** (**acc.no NM013261.3**): AAAGGATGCGCTCTCGTTCA, R: TCTACTGCCTGGAGACCTTGATC; **IL-6** (**acc_no NM000600**): F: CGGGAACGAAAGAGAAGCTCTAT, R: AGGCGCTTGTGGAGAAGGA; **PDK4** (**acc_no BC040239**): F: TTTCCAGACCAACCAATTCACA, R: TGCCCGCATTGCATTCTTA; **GLUT1** (**acc_no K03195**): F: CAGCAGCCCTAAGGATCTTCTCA, R: CCGGCTCGGCTGACATC; **GLUT4** (**acc_no M20747**): F: ACCCTGGTCCTTGCTGTGTT, R: ACCCCAATGTTGTACCCAAACT; **MHCI** (**acc_no NM005963**) : F: CCAGACTGTGTCTGCTCTCTTCAG, R: CAGGACAAGCTCATGCTCC-AT; **MHCIIa** (**acc_no NM017534**): F: AAGGTCGGCAATGAGTATGTCA, R: CAACCATCCACAGGAACATCTTC.

### Data presentation and statistics

Statistical analysis of the overall effects of acute, high-frequency EPS on the contents of ATP, PCr and lactate was performed using linear mixed models (LMM) (SPSS version 17, SPSS Inc., Chicago, IL). In experiments where effects of 24 h and 48 h EPS were compared to unstimulated control cells, data were analyzed using non-parametric Kruskal-Wallis test, while in experiments where two groups were compared (EPS-treatment versus unstimulated control cells), non-parametric Wilcoxon matched pair tests were performed (GraphPad Prism 5.0 for Windows, GraphPad Software Inc., San Diego, CA). All values in figures are presented as means±SEM, with n representing the number of experiments performed, each experiment were performed with cells from separate (different) donors, with triplicate samples in each experiment. Statistical significance was set at *P*<0.05. In most experiments, results are presented normalized to unstimulated control cells, and the absolute values of unstimulated control cells are stated in the figure text.

## Results

### Effects of acute, high-frequency EPS on deoxyglucose uptake, cell contents of ATP, PCr and lactate in cultured myotubes

**Figure 1:**
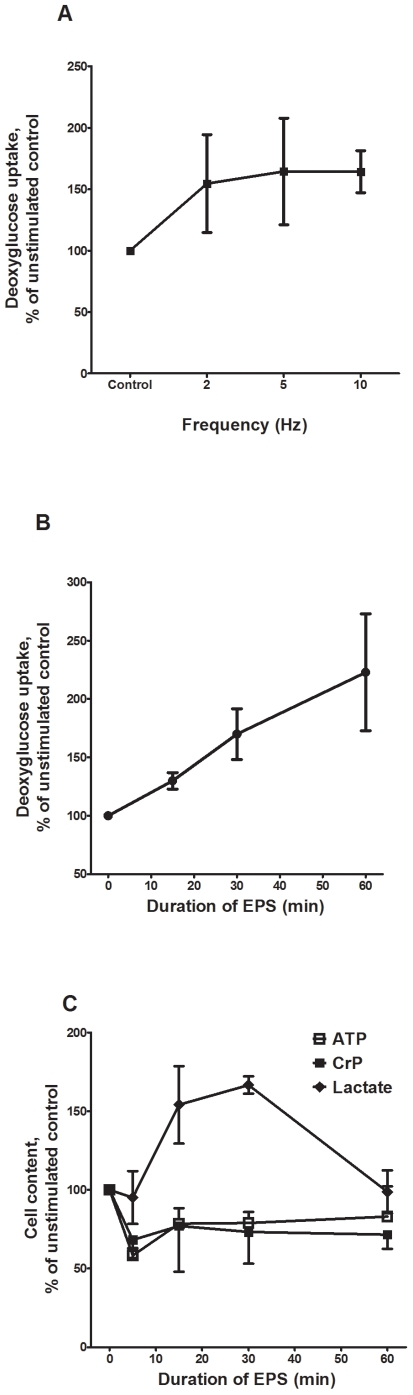
Frequency-dependence of deoxyglucose uptake (**A**) **effects of acute, high-frequency EPS on deoxyglucose uptake** (**B**) **and cell contents of ATP, PCr and lactate** (**C**)**.** A: Frequency-dependence of deoxyglucose uptake: Eight days after the onset of differentiation, cultured myotubes were incubated in serum-free DMEM (5.5 mmol/L glucose) for 1 h in a 5% CO_2_ incubator at 37°C, before addition of [^3^H]deoxyglucose (1 vµCi/mL). Electrical pulse stimulation (30 V, pulse trains of 200 ms) was applied to the cells using frequencies of either 2, 5 or 10 Hz for the first 15 min of the deoxyglucose uptake period (60 min). Values are presented as means±SEM of 4 experiments, normalized to unstimulated control cells (absolute values 56.4–333.6 nmol/mg). B:Deoxyglucose uptake: Eight days after the onset of differentiation period, cultured myotubes were incubated in serum-free DMEM (5.5 mmol/L glucose) for 1 h in a 5% CO_2_ incubator at 37°C, before addition of [^3^H]deoxyglucose (1 µCi/mL). Electrical pulse stimulation (100 Hz, 30 V, pulse trains of 200 ms given every 5^th^ second) was applied to the cells the first 5-15-30 min of the deoxyglucose uptake period or during the whole period (60 min) of deoxyglucose uptake. Values are presented as means±SEM of 6 experiments, normalized to unstimulated control cells (57.9–92.5 nmol/mg). C: Cell contents of ATP, phosphocreatine (PCr) and lactate: Eight days after the onset of differentiation period, the myotubes were preincubated for 1 h (37°C, 5% CO_2_) with DMEM and high-frequency electrical stimulation (100 Hz, 30 V, pulse trains of 200 ms given every 5^th^ second) was applied for 5–60 min. The cells were analyzed for ATP and PCr levels with luminescence spectrometry, and cell content of lactate was analyzed with fluorescence spectrometry as described in Materials and Methods. Values are presented as means±SEM of 3 experiments, normalized to unstimulated control cells (absolute values: ATP; 42.5–73.9 nmol/mg, PCr; 48.4–169.1 nmol/mg and lactate; 1.9–87.6 nmol/mg). Overall effect of electrical pulse stimulation on cells was statistically significant (linear mixed model, SPSS) compared to unstimulated control cells (*P = *0.001 for ATP, *P = *0.007 for PCr and *P = *0.03 for lactate).

To verify that electrical stimulation of cultured human myotubes leads to expected metabolic changes, the myotubes were exposed to acute, high-frequency EPS, and deoxyglucose uptake and cellular contents of ATP, PCr and lactate were examined. Frequency-dependence of deoxyglucose uptake during EPS is showed in Figure 1A. Acute, high-frequency EPS increased deoxyglucose uptake in cultured myotubes (Fig. 1B). This uptake was specific, since it was inhibited by cytochalasin B (20 µM) (data not shown). In addition, the amount of deoxyglucose taken up by electrically stimulated myotubes correlated positively with the duration of stimulation. Cell contents of ATP and PCr in electrically stimulated myotubes were compared to unstimulated control cells incubated for the same period of time. In response to 5–60 min of electrical stimulation, the contents of both ATP and PCr decreased significantly (*P = *0.001 and *P = *0.007, respectively), while the amount of lactate significantly increased (*P = *0.03) (Figure 1C, overall effect, linear mixed model, SPSS). Together, these findings indicated that the cells were contracting and consuming energy, and that they responded to acute EPS in a similar way as a single bout of exercise *in vivo.*

### Effects of chronic, low-frequency EPS on glucose metabolism in cultured human skeletal muscle cells

We were further interested in whether the effects of chronic, low-frequency EPS on cultured human skeletal cells could mimic the effects of regular exercise *in vivo*. A movie showing contractions of cultured skeletal muscle cells under EPS is attached as Video S1.

**Figure 2:**
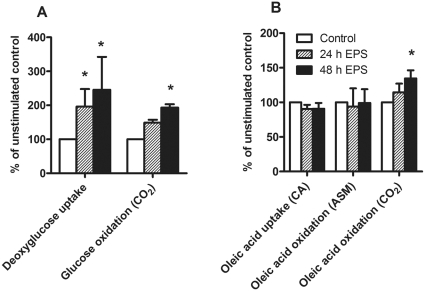
Effects of chronic, low-frequency EPS on glucose (**A**) **and oleic acid metabolism** (**B**)**.** Cultured myotubes were electrically stimulated (1 Hz, 2 ms pulses, 30 V), for the last 24 h or 48 h of the differentiation period. (A) **Deoxyglucose uptake**: After termination of electrical stimulation (day 8 of differentiation), uptake of [^3^H]deoxyglucose (1 µCi/mL) was measured for 1 h as described in Materials and Methods. Values are presented as means±SEM of 6 experiments. *Significantly different from unstimulated control cells (absolute values 28.0–170.5 nmol/mg protein) (*P = *0.004, non-parametric Kruskall-Wallis test). **Glucose oxidation**: After termination of electrical stimulation (day 8 of differentiation), the rate of D-[^14^C(U)]glucose (2 µCi/mL) oxidation was measured as described in Materials and Methods. Values are presented as means±SEM of 4 experiments. ^*^Statistically significant compared to unstimulated control cells (absolute values 2.7–28.4 nmol/mg protein) (*P = *0.008, non-parametric Kruskall-Wallis test). (B) **Oleic acid metabolism**: Eight or nine days after the onset of differentiation, myotubes were exposed to [1-^14^C]OA (1 µCi/mL) for 2 h, and CO_2_, ASMs and cell-associated (CA) radioactivity were measured as described in Materials and Methods. Values are presented as means±SEM of 8 experiments. *Statistically significant vs. unstimulated control myotubes (absolute values 15.0–166.8 nmol/mg protein for CA, 0.6–4.0 nmol/mg protein for ASMs and 0.2–2.9 nmol/mg protein for CO_2_) (*P = *0.04, non-parametric Kruskall-Wallis test).

Deoxyglucose uptake was significantly increased (*P = *0.004) in cultured myotubes after both 24 and 48 h of chronic, low-frequency EPS by 96% and 145%, respectively, compared to unstimulated control cells. The insulin effect was unaffected by EPS (data not shown). As with acute, high-frequency EPS, the observed increase in deoxyglucose uptake after chronic, low-frequency EPS was specific, since it was inhibited by cytochalasin B (20 µM) (data not shown). Glucose oxidation, measured as the amount of CO_2_ produced, was also significantly increased (*P = *0.008) after 48 h of chronic, low-frequency EPS compared to unstimulated control (Fig. 2A).

### Effects of chronic, low-frequency EPS on fatty acid metabolism in cultured myotubes

A known effect of exercise is to increase oxidative capacity of the cells, resulting in increased oxidation of both glucose and fatty acids. We examined the effect of chronic, low-frequency EPS on oleic acid metabolism by measuring uptake, and production of acid soluble metabolites (ASMs) and CO_2_. Complete oleic acid oxidation, measured as the amount of produced CO_2_, was significantly increased (*P = *0.04) after 48 h of EPS by 35% compared to control cells (Fig. 2B). Uptake of oleic acid was unaffected by EPS compared to unstimulated control cells (Fig. 2B). β-oxidation, measured as the amount of ASMs, also remained unchanged after EPS compared to unstimulated control cells (Fig. 2B).

### Effects of chronic, low-frequency EPS on mitochondrial content and citrate synthase activity

**Figure 3:**
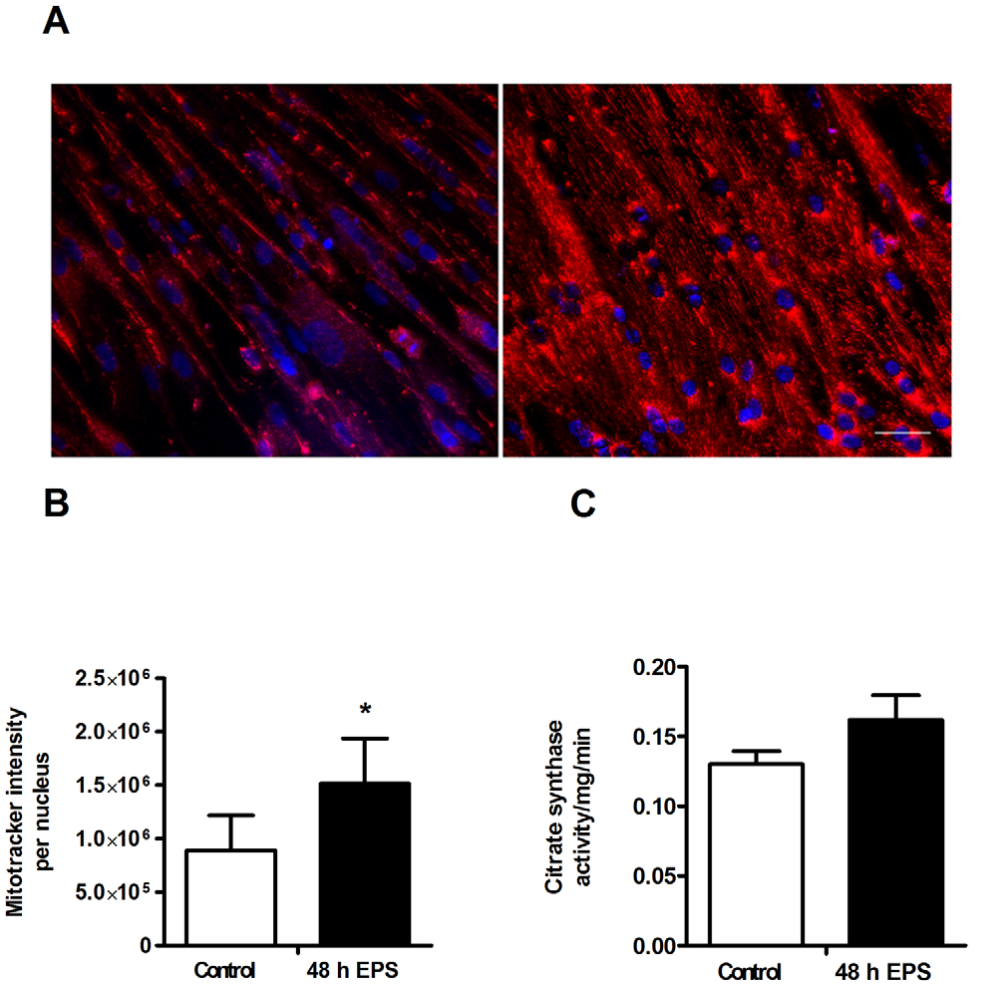
Effects of 48 h of chronic, low-frequency EPS on mitochondrial content and citrate synthase activity. Low-frequency EPS was applied to cultured myotubes for the last 48 h of the eight days differentiation period as described in Materials and Methods. (**A**) **Live imaging of mitochondria:** The cells were stained for nuclei (blue) and mitochondria (red) as described in Materials and Methods. Scale bar is 50 µm. Left: Unstimulated control myotubes. Right: Myotubes after 48 h of chronic, low-frequency EPS. (**B**) **Mitochondrial content in electrically stimulated myotubes**: Mitochondrial content was measured by live imaging after 48 h of chronic, low-frequency EPS. Values are presented as means±SEM of 6 experiments. *Statistically significant vs. unstimulated control (*P = *0.03, non-parametric Wilcoxon matched pair test). (**C**) **Citrate synthase activity in electrically stimulated myotubes:** Enzyme activity was determined spectrophotometrically from cell homogenates prepared from the myotubes after 48 h of chronic, low-frequency EPS as described in Materials and Methods, and compared to activity in unstimulated control cells. Values are presented as means±SEM of 5 experiments.

To support the results obtained in the metabolic experiments with glucose and oleic acid, we investigated effects of chronic, low-frequency stimulation for 48 h on mitochondrial content in cultured myotubes. Representative images are presented for control, unstimulated myotubes in figure 3A (left), and for electrically stimulated myotubes in figure 3A (right). Total intensity of MitoTracker®Red FM per nucleus was significantly increased (2.2-fold, Fig. 3B, *P* *= *0.03), and the citrate synthase activity tended to increase (*P* = 0.1, Fig. 3C) in EPS-treated cells compared to unstimulated control cells.

### Effects of chronic, low-frequency EPS on gene and protein expression in cultured myotubes

**Figure 4:**
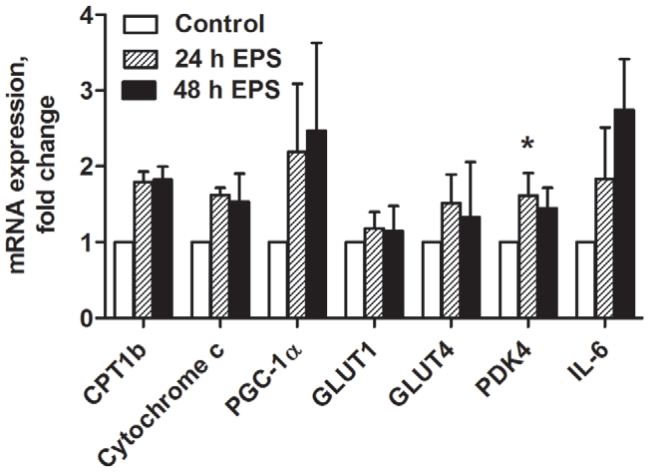
Effects of chronic, low-frequency EPS on gene expression. Low-frequency EPS was applied to cultured myotubes for the last 24 h or 48 h of the eight days differentiation period. mRNA was isolated and expression assessed by real time RT-PCR as described in Materials and Methods, and values are presented as means±SEM of 3–6 experiments, normalized to levels of housekeeping genes 36B4. The ranges of the fold changes of the mRNA expression levels in the control groups normalized to the level of housekeeping gene 36B4 were as follows: 0.3–1.9 for CPT1b, 0.4–1.3 for cytochrome C, 0.001–0.6 for PGC-1α, 0.6–1.2 for GLUT1, 0.2–2.6 for GLUT4, 0.7–1.0 for PDK4 and 0.1–1.1 for IL-6. *Statistically significant vs. unstimulated control cells (*P = *0.04, non-parametric Kruskall-Wallis test).

The observed functional changes in fatty acid and glucose metabolism were accompanied by a range of changes in mRNA expressions (Fig. 4). mRNA expression level of pyruvate dehydrogenase complex kinase 4 (PDK4) was significantly increased (*P = *0.04) after 24 h of EPS (Fig. 4). mRNA expression levels of following genes also tended to increase: CPT1b (*P = *0.06), Cytochrome c (*P = *0.07), PGC-1α (*P = *0.2) and IL-6; a myokine reported to be secreted from contracting skeletal muscles *in vivo* [35], [36] (*P = *0.07) (Fig. 4). GLUT1 and GLUT4 were not affected by EPS (Fig. 4). The results from immunoblots of phosphorylated Akt to total Akt ratio, showed no effect of electrical pulse stimulation (data not shown). Protein expressions of the complexes in the electron transport chain were unchanged by EPS (data not shown).

### Effects of chronic, low-frequency EPS on the markers of slow-oxidative **(**MHCI**)** and fast glycolytic **(**MHCIIa**)** fiber type

**Figure 5:**
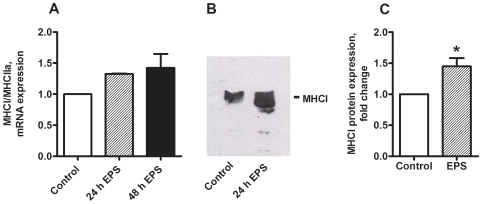
Effects of chronic, low-frequency EPS on markers of slow-oxidative (**MHCI**) **and fast-glycolytic** (**MHCIIa**) **muscle fiber types.** Low-frequency EPS was applied to cultured myotubes for the last 24 h or 48 h of the eight days differentiation period as described in Materials and Methods before the cells were harvested. (**A**) **MHCI/MHCIIa mRNA ratio:** mRNA was isolated from cultured myotubes after the EPS treatment. Expression was assessed by RT-PCR as described in Materials and Methods, and values are presented as means±SEM of 4 experiments, normalized to levels of housekeeping genes 36B4. (**B**) (**C) Immunoblot analysis of MHCI after 24**–**48** **h of EPS:** Aliquots of 40 µg cell protein from total cell lysates prepared in Laemmli buffer were electrophoretically separated on NuPAGE® 4–12% (w/v) Bis-Tris Gel, followed by immunoblotting with specific antibody for slow-oxidative MHCI. (B) One representative immunoblot. (C) Densitometric analysis of immunoblots, values are presented as means±SEM of 6 experiments. *Statistically significant vs. unstimulated control cells (*P = *0.03, non-parametric Wilcoxon matched pair test).

To assess the effect of EPS on fiber type markers in myotubes, we further investigated expression of genes and proteins specifically enriched in either type I (slow) fibers (MHCI) or type IIa (fast) fibers (MHCIIa). Expressions of MHCI and MHCIIa appeared to increase and decrease, respectively, thus the MHCI/MHCIIa mRNA ratio tended to increase (*P = *0.06) in electrically stimulated myotubes (Fig. 5A). Protein expression of MHCI was significantly increased (*P = *0.03) by 45% after 24–48 h of EPS (Fig. 5B, C).

### Evaluation of toxic effects of EPS on cultured myotubes

Protein contents of the cells were unaffected by acute, high-frequency as well as chronic, low-frequency EPS under the conditions used in the experiments (data not shown). Further, lactate dehydrogenase content was unchanged in media from myotubes exposed to chronic, low-frequency EPS for both 24 h and 48 h compared to unstimulated control cells (data not shown). Number of nuclei in myotubes after 48 h of chronic, low-frequency EPS, determined by live imaging of the cells, did not differ from unstimulated control cells (data not shown). After staining with trypan blue, a low percentage (less than 1%) was stained, both in control cells and in electrically stimulated myotubes, with no difference between the two groups (data not shown). In conclusion, EPS did not induce toxic effects to cultured human skeletal muscle cells.

## Discussion

Cultured myotubes are a valuable tool for investigation of metabolic processes in skeletal muscles. In the present study, we demonstrate how a cell model of human myotubes can be established as an *in vitro* model of exercise, which can be used to study some of the adaptations seen in trained skeletal muscles. Two different patterns of electrical pulse stimulation (EPS) were applied: acute, high-frequency EPS (bipolar pulse trains of 200 ms, 100 Hz, given every 5^th^ second, 30 V for 5–60 min) to simulate a single bout of exercise; and chronic, low-frequency EPS (single, bipolar pulses of 2 ms, 1 Hz at 30 V for 24 or 48 h) to simulate regular exercise.

By acutely stimulating cultured myotubes with high-frequency EPS, glucose uptake and cell lactate content increased, while ATP and PCr contents decreased. By continuously applying chronic, low-frequency EPS, we successfully increased oxidative capacity of the cells by increasing glucose metabolism and complete oleic acid oxidation. Further, these functional changes in the metabolic processes were accompanied by doubling of mitochondrial content, measured as total intensity of MitoTracker®Red, after 48 h of EPS. Citrate synthase activity tended to increase in EPS-treated myotubes, although not significantly. Increased mitochondrial content in skeletal muscles after exercise is believed to result from the cumulative effects of transient increases in mRNA transcripts encoding mitochondrial proteins after repeated exercise sessions [3], [47]. Temporal sequences of molecular effects that occur in human muscle when mitochondrial biogenesis is induced with exercise training were investigated in a study by Perry et al. [48]. Although CS mRNA increased already after first training session, increase in the activity of CS was not observed until the end of the 3^rd^ of the seven performed training sessions. In general, repeated transient bursts of mRNA were shown to occur in the early phases of training, before increases in the activities of mitochondrial proteins, but the time and magnitude of mRNA and protein responses of different transcriptional and mitochondrial proteins also showed considerable variation, depending on the phase of the exercise they were measured in [48]. Thus, the lack of significant increase in CS activity in our experiments after only 48 hours of EPS is in agreement, or at least reflects the complexity and precise time-dependence of molecular events that were described to occur in mitochondrial biogenesis during exercise in human skeletal muscles. At applied conditions, we did show expected functional changes (lipid oxidation and glucose metabolism), as well as changes in mRNA and protein expressions of some factors, but in order to demonstrate other changes, both on mRNA and protein level, we would perhaps have to use different patterns and time periods, and with the present model, this is something that can be done in the future work. The same inconsistency between citrate synthase activity and MitoTracker®Red intensity has also been reported in a recent work presenting a novel, exercise-mimicking approach to remodel lipid metabolism in cultured human myotubes [49]. Interestingly, this was a cell system like ours, but the exercise-mimicking effects were induced by a pharmacological activation. Thus, for future studies, time aspect may be an important factor to be considered when performing exercise-mimicking studies in cell cultures.

Changes in expression levels of a range of genes were also observed after chronic EPS. mRNA level of PDK4 was significantly increased in electrically stimulated cells, while mRNA levels of Cytochrome c and CPT1b also tended to increase. GLUT1 and GLUT4 did not appear to be affected by EPS. Moreover, the ratio of the mRNA level of MHCI (a gene marker of type I, slow oxidative fiber type), to that of MHCIIa (a gene marker of glycolytic, fast-twitch skeletal muscle fibers) tended to increase in electrically stimulated myotubes, and this finding was also supported by increased protein expression of MHCI in EPS-treated cells. Further, mRNA expression of peroxisome proliferator-activated receptor γ coactivator-1α (PGC-1α), a transcriptional cofactor referred to as the master regulator of mitochondrial function and biogenesis [50]; which is frequently considered as an important factor in cellular mechanisms evoked by exercise, tended to increase, although not significantly, as well as mRNA level of IL-6, an interleukin known to be secreted by skeletal muscles after exercise *in vivo*, strengthening the conclusion that our model of EPS may resemble trained muscle.

The relative contribution of fatty acid oxidation to total fuel demand is increased in healthy subjects performing moderate-intensity exercise, and several studies support that exercise reduces the reliance on carbohydrates as an energy source and increases fatty acid oxidation [51], [52]. The rate of oleic acid oxidation was significantly increased after 48 h of EPS in our cell culture model, while the uptake of oleic acid was unchanged. Although exercise has been shown to increase uptake of fatty acids in humans, cellular mechanisms of elevated uptake are still not clear, since inconsistencies exist due to different duration and intensity of training studies [53]. A key factor facilitating transport of fatty acids through the carnitine shuttle over the outer mitochondrial membrane, CPT1b, has been shown to be increased in an *in vivo* human training study with moderate-intensity exercise for a shorter duration (2 months), accompanied by an increase in mitochondrial fatty acid oxidation rate [54]. Although not significantly, mRNA expression level of CPT1b tended to increase in our EPS cell model as well.

Our model of chronic, low-frequency EPS showed both increased import and oxidative metabolism of glucose, and these effects are also known from *in vivo* trained fibers [25], [26]. However, we did not observe any additional effect of insulin and EPS on glucose uptake, nor was phosphorylation of Akt affected by EPS. These observations are in agreement with suggestions of an insulin-independent pathway to enhance glucose uptake [55], [56]. In addition, there are proposals that some key metabolic substances typically triggered by insulin, may also be activated during muscle contractions in the absence of this hormone [26]. mRNA expression levels of GLUT1 and GLUT4 were unaffected by EPS. GLUT4 is often deficient in cultured skeletal muscle cells [57], and in primary human myotubes, basal glucose uptake is generally mediated by other glucose transporters, such as GLUT1 and GLUT3 [58], [59]. Inconsistencies between mRNA levels of GLUTs and functional data have previously been reported in cultured human skeletal muscle cells [60], [61]. On the other hand, PDK4, an inhibitor of pyruvate dehydrogenase complex, which is an important factor in switching oxidation towards fatty acids [62], was significantly increased in EPS-treated cells, indicating a possible switch in the fuel preference of the myotubes. When grown in culture, satellite cells mature to myotubes that generally display the characteristics of glycolytic type II muscle fibers [41], and are characterized by low mitochondrial oxidative capacity [63], [64], with higher fuel preference for carbohydrates over lipids [65]. This could be due to lack of proliferation of mitochondria *in vitro* in the absence of appropriate environmental signals, such as contractions. Thus, approaches that increase mitochondrial oxidative potential of human myotubes are highly relevant with respect to studies on cellular energy metabolism.

Even though it is difficult to directly compare effects of *in vivo* exercise to the observed effects in our model of EPS in cultured human myotubes, several of our observations display important aspects of the *in vivo* effects of exercise. In summary, by applying our model of chronic continuous, low-frequency EPS, we observed important functional changes in cell culture: improved lipid oxidation and glucose metabolism, which are known effects of exercise *in vivo*. Further, we also demonstrated a possible fiber-type switch, measured by increased protein expression of MHCI in EPS-treated cells. To our knowledge, such changes have not previously been reported in human cell cultures. Thus, we believe that our model of EPS in cultured human skeletal muscle cells represents a unique, physiologically relevant *ex vivo* model, which can be used to further study interrelationship between exercise-induced cellular mechanisms and underlying signalling pathways under controlled conditions. Particularly, the present model might be of great interest in clarifying the potential of contractions on energy metabolism in skeletal muscle cells obtained from different groups of individuals (obese, glucose intolerant, athletes etc.). Currently, contraction-induced effects on energy metabolism in human skeletal muscle cells originating from extremely obese individuals with or without type 2 diabetes are being investigated.

## Supporting Information

Video S1 **A film showing contractions of cultured human skeletal muscle cells exposed to chronic, low-frequency EPS.** Cultured human skeletal muscle cells were exposed to chronic, low-frequency EPS (single, bipolar pulses of 2 ms, with 30 V and 1 Hz continuously for the last 24 or 48 h of differentiation period). The observed contractions were synchronous with the electrical pulses.
(M4V)
