# Correction: Electrical Pulse Stimulation of Cultured Human Skeletal Muscle Cells as an *In Vitro* Model of Exercise

**DOI:** 10.1371/annotation/270b432d-50ec-41f1-ad4d-ddd9f51f62a5

**Published:** 2013-03-04

**Authors:** Nataša Nikolić, Siril Skaret Bakke, Eili Tranheim Kase, Ida Rudberg, Ingeborg Flo Halle, Arild C. Rustan, G. Hege Thoresen, Vigdis Aas

There was a mistake in Figure 5A. The primer used for mRNA expression was actually MYH1 which regulates expression of MHC type IIx muscle fibers and not MHC type I as stated in the figure. However, the authors have repeated the experiment with the correct primer MYH7 (acc_no NM000257.2, F: CTCTGCACAGGGAAAATCTGAA, R: CCCCTGGAGACTTTGTCTCATT), and the new Figure 5 is shown below. This makes no difference to the conclusions of the paper. Chronic low frequent electrical pulse stimulation for 48 h increased expression of slow muscle fibers assessed by Western blotting, but there were no significant change in mRNA expression observed at the same time, neither was the MHCI/MHCIIa mRNA ratio significantly increased (P=0.125, non-parametric Wilcoxon matched pair test, n=4).

The correct image of Figure 5 can be seen here: 

**Figure:**
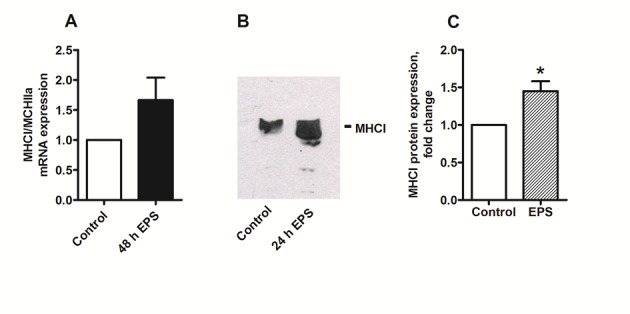


The correct legend for Figure 5 is: Effects of chronic, low-frequency EPS on markers of slow-oxidative (MHCI) and fast-glycolytic (MHCIIa) muscle fiber types. Low-frequency EPS was applied to cultured myotubes for the last 24 h or 48 h of the eight days differentiation period as described in Materials and Methods before the cells were harvested. (A) MHCI/MHCIIa mRNA ratio: mRNA was isolated from cultured myotubes after the EPS treatment. Expression was assessed by RT-PCR as described in Materials and Methods, and values are presented as means ± SEM of 4 experiments, normalized to levels of housekeeping genes 36B4. (B) (C) Immunoblot analysis of MHCI after 24–48 h of EPS: Aliquots of 40 µg cell protein from total cell lysates prepared in Laemmli buffer were electrophoretically separated on NuPAGE® 4–12% (w/v) Bis-Tris Gel, followed by immunoblotting with specific antibody for slow-oxidative MHCI. (B) One representative immunoblot. (C) Densitometric analysis of immunoblots, values are presented as means±SEM of 6 experiments. *Statistically significant vs. unstimulated control cells (P = 0.03, non-parametric Wilcoxon matched pair test). 

